# Elevation of circulating TNF receptor 2 in cancer: A systematic meta-analysis for its potential as a diagnostic cancer biomarker

**DOI:** 10.3389/fimmu.2022.918254

**Published:** 2022-11-16

**Authors:** Apriliana E. R. Kartikasari, Emily Cassar, Mohammed A. M. Razqan, Crispin Szydzik, Cesar S. Huertas, Arnan Mitchell, Magdalena Plebanski

**Affiliations:** ^1^ Translational Immunology and Nanotechnology Theme, School of Health and Biomedical Sciences, Royal Melbourne Institute of Technology (RMIT) University, Bundoora, VIC, Australia; ^2^ Integrated Photonics and Applications Centre (InPaC), School of Engineering, Royal Melbourne Institute of Technology (RMIT) University, Melbourne, VIC, Australia

**Keywords:** circulating biomarker, cancer, sTNFR2, diagnosis, prognosis

## Abstract

High Tumor Necrosis Factor Receptor 2 (TNFR2) expression is characteristic of diverse malignant cells during tumorigenesis. The protein is also expressed by many immunosuppressive cells during cancer development, allowing cancer immune escape. A growing body of evidence further suggests a correlation between the circulating form of this protein and cancer development. Here we conducted a systematic meta-analysis of cancer studies published up until 1^st^ October 2022, in which the circulating soluble TNFR2 (sTNFR2) concentrations in patients with cancers were recorded and their association with cancer risk was assessed. Of the 14,615 identified articles, 44 studies provided data on the correlation between cancer risk and the level of circulating sTNFR2. The pooled means comparison showed a consistently significant increase in the levels of sTNFR2 in diverse cancers when compared to healthy controls. These included colorectal cancer, ovarian cancer, breast cancer, non-Hodgkin’s lymphoma, Hodgkin’s lymphoma, lung cancer, hepatocarcinoma, and glioblastoma. In a random-effect meta-analysis, the cancer-specific odd ratios (OR) showed significant correlations between increased circulating sTNFR2 levels and the risk of colorectal cancer, non-Hodgkin’s lymphoma, and hepatocarcinoma at 1.59 (95% CI:1.20-2.11), 1.98 (95% CI:1.49-2.64) and 4.32 (95% CI:2.25-8.31) respectively. The overall result showed an association between circulating levels of sTNFR2 and the risk of developing cancer at 1.76 (95% CI:1.53-2.02). This meta-analysis supports sTNFR2 as a potential diagnostic biomarker for cancer, albeit with different predictive strengths for different cancer types. This is consistent with a potential key role for TNFR2 involvement in cancer development.

## Introduction

Cancer remains one of the most lethal diseases and is currently the world’s second most common cause of death (1). According to the World Health Organization, approximately 9.6 million deaths occur annually because of cancer (1). Currently, the high mortality rate from cancers is mostly due to late diagnosis as many cancers can be efficiently treated if diagnosed early. The existing cancer diagnostics methods and techniques present various limitations. While diagnostic imaging techniques such as digital mammography, ultrasonography, computed tomography, and magnetic resonance imaging are non-invasive, they lack absolute sensitivity and specificity for the detection of different cancer types (2). Furthermore, these imaging techniques require expensive specialized equipment and highly trained medical personnel, which limits patient access due to high costs (3). On the other hand, while biopsy staining is useful for definitive cancer diagnosis, its invasive nature makes it unattractive for most patients and it is less sensitive to early-stage cancers. Thus, diagnostic biomarkers from a minimally invasive liquid biopsy such as blood that could identify the presence of specific cancers with high precision and at their early stage, are highly desired.

Tumor necrosis factor (TNF) is a cytokine implicated in inflammation and cancer development (4–6). In the tumor microenvironment, TNF *via* its receptor TNFR1 and TNFR2 plays a dual role to suppress or promote cancer proliferation and metastasis (7, 8). TNFR1 can be expressed by nearly all cells, while TNFR2 can be highly expressed by tumor cells (9–11). In malignant cells, TNFR2 promotes tumor cell proliferation and is increasingly being considered as an oncogene as it is overexpressed in more than 20 types of cancer, including multiple myeloma, human renal cell carcinoma, breast, oesophageal, myeloma, colon cancer, ovarian cancer, and cutaneous T-cell lymphomas, among others (9–11). The presence of TNFR2 at cancer sites has prompted research on utilizing TNFR2 as a target for therapeutic agents.

TNFR2 is also highly expressed in immune cells, which could be associated with tumorigenesis and tumor growth or conversely tumor controls (10, 12, 13). This protein however has been shown to be broadly expressed in the repertoire of immunosuppressive cells present on tumors and tumor microenvironments promoting pro-tumor activity (9). They include regulatory T cells (Tregs) (14, 15), natural killer cells (NK cells) (16), myeloid-derived suppressor cells (MDSCs) (17), mesenchymal stem cells (MSCs) (18, 19), endothelial progenitor cells (EPCs) (20), neural stem cells (NSCs) (21) and cancer-associated fibroblasts (CAFs) (22). The immunosuppressive cells are activated by the TNF-TNFR2 axis as well as the TNFR2 alone without its ligand, as TNFR2 can auto-associate in the absence of TNF and promote active signaling (23). The active immunosuppressive cells could then promote cancer immune evasion by suppressing the immune response against cancer. TNFR2 overexpression and TNF-TNFR2 signaling on Tregs results in their proliferation into a subpopulation of highly suppressive phenotype, promoting enhanced immunosuppressive activities within tumor microenvironments (24) which in turn promotes tumor cell proliferation (25). As an example, the expression of TNFR2 by Tregs in peripheral blood is strongly correlated with cancer development of the lymphatic system (lymph nodes), distant metastases, and advanced lung cancer disease (26). In NK cells, the TNF-TNFR2 axis acts as a checkpoint molecule, reducing NK cells’ tumoricidal activity (16). In MDSCs, TNFR2 boosts differentiation capacity and immunosuppressive activity of these immunosuppressive cells as well as promoting the activation of Tregs (27). TNFR2 promotes MDSC survival by inhibiting the apoptosis processes of the cells, which in turn contributes to tumorigenesis (12, 28). Similarly, in MSCs, EPCs, and NPCs, the TNF-TNFR2 axis promotes immunosuppression within the tumor microenvironment and induction of active Tregs (18, 20, 21, 29). In addition, the TNF-TNFR2 signaling on CAFs enhances the synthesis of immunosuppressive interleukin (IL)-33, which increases tumor cell migration and invasion (22). On the contrary, TNFR2 is an important costimulatory molecule to enhance the proliferation and activation of both CD4^+^ and CD8^+^ conventional effector T cells (Teffs) necessary to eliminate the neoplastic cells (14, 30, 31).

The levels of circulating form of TNFR2 (sTNFR2) has been shown to increase in chronic inflammatory conditions such as obesity and Type 2 diabetes (DM2), diabetic kidney disease characterized by increased albuminuria, and juvenile chronic arthritis (32–35), as well as in infectious diseases including severe malaria (36). The circulating sTNFR2 comes from membrane shedding or as a spliced variant, following immune cell activation (37–41). This soluble receptor is secreted *in vivo* by Tregs and consistently counteract the action of TNF (42), which in turn suppresses the active immune response exerted *via* TNFR1, and meanwhile, the membrane-bound TNFR2 on Tregs can independently promote immunosuppressive behavior of Tregs (14, 15). *In vitro*, some pathogens stimulate the secretion of sTNFR2 (43, 44). *In vivo*, TNFR2-overexpressing cancer cells promote the accumulation of TNFR2^+^ Tregs in the draining lymph nodes and increase the levels of sTNFR2 in the circulation (45). These studies suggest that elevated sTNFR2 could be an indicator of cancer (10, 12, 13). Therefore, the circulating sTNFR2 could potentially lend itself as a novel diagnostic biomarker to detect the presence of cancer. Thus, we conducted a systematic review and meta-analysis to test the utility of sTNFR2 as a diagnostic biomarker for cancer.

## Materials and methods

### Study design

The study was designed to evaluate the utility of sTNFR2 in plasma or serum as a diagnostic biomarker for various cancers and a prognostic biomarker to predict cancer outcome, by performing a meta-analysis on published studies. The literature research was conducted by the authors following the guideline set by the statements provided by “Preferred Reporting Items for Systemic Reviews and Meta-Analysis” (PRISMA) (46).

### Search strategy

The literature was searched systematically in Medline, Embase, and Scopus databases, from inception to 1^st^ October 2022, for studies investigating the associations between circulating sTNFR2 and cancer. The text word search included (TNFR2, TNFR2-p75, TNFR2p75, TNFRp75, TNFR-p75, sTNFR2 or CD120b) and (cancer, cancers, carcinoma, tumor, neoplasm, malignant, or malignancy). Duplicates were removed using EndNote20 software (Clarivate Analytics, Boston, USA), and this software was further used to select articles.

### Inclusion and exclusion criteria

The selection of articles for studies was based on defined inclusion and exclusion criteria. Titles, abstracts, and full articles were first screened independently by three authors (AK, EC, MR). Case studies, conference papers, animal and *in vitro* studies were excluded. Secondary source articles such as meta-analyses and reviews that do not provide the original data of a study were excluded. No restriction to time or age was applied. Additional search by scanning the reference lists from other related articles was also performed. Relevant articles were then independently reviewed by the three authors (AK, EC, MR) and selected based on the content of the articles, which includes: 1. the study is on cancer, 2. the biomarker of interest is soluble TNFR2 in serum or plasma. The collected articles were sorted and recorded using the PRISMA flow diagram (46).

### Quality assessment

The article’s quality was assured by noting the author, year, abstract, and number of citations. Importantly, the study fitness was assessed by two authors (AK, EC) in discussion, using the updated Strengthening the Reporting of Observational studies in Epidemiology (STROBE) checklist for diagnostic studies (47).

### Data collection and extraction

Three reviewers (AK, EC, and MR) recorded data from all studies that met inclusion criteria using a standardized data collection procedure. Data collected was arranged in a table for all the studies (**Supplementary Table 1**). Study title, author’s name, name of journal, year, cancer type, country of study, the biological liquid used, population ethnicity, age, and gender were recorded. The number of patients and healthy controls participating in the study were recorded. Furthermore, the reported values related to sTNFR2 levels in serum or plasma were recorded. Additionally, the odds ratios presented in the studies were recorded or calculated based on the provided supporting data available in the studies.

### Data analysis and statistics

The changes in the levels of sTNFR2 levels in various cancers were interrogated by determining the pooled mean values of sTNFR2 concentrations in serum/plasma from the controls and cancer patients. The pooled mean value is the mean of the weighted means from the selected studies, obtained by log-transforming the means as the sTNFR2 is expected to display log beta distribution and adjusting the means with their corresponding sample sizes (48). We also constructed the 95% confidence interval (CI) following log-transformed data. When median values were recorded, we estimated the mean values following Cochrane recommendations and the handbook (49)with the calculation described by Wan et al. (2014) (50). Then, we applied the Tukey test to assess the significant difference of sTNFR2 levels in cancer in comparison to controls. We then back-transformed the variables that have been log transformed for reporting purposes.

To evaluate the diagnostic utility of sTNFR2, we extracted odd ratio values from the selected studies, as this style of meta-analysis is frequently used to quantify disease occurrence in populations to answer concerns about disease risk (51). Since there is variability in study populations, especially with differs cancer types, the methodology of sample handling, as well as the methodology of sTNFR2 measurements, we utilized a random-effects meta-analysis model to evaluate the sTNFR2 correlation with the risk of cancer in our selected studies (52). We then generated the forest plot using Review Manager 5.4, London, UK, that calculated the overall odds ratios for each cancer type, and all cancers combined. An odds ratio of more than one with a P-value of <0.05 indicates that there is a likelihood of cancer occuring and a potential for sTNFR2 to indicate the presence of cancer. The between-study invariance in our random-effect meta-analysis was calculated using τ^2^, as the estimated standard deviation of underlying effects across included studies (49). The Chi^2^ and I^2^ tests measure the heterogeneity between studies. I^2^ above 50% could represent substantial heterogeneity depending on the magnitude and direction of the effects and the P-value of the Chi^2^ test (49). Publication bias was assessed based on the presence of data asymmetry on the Funnel plot (49).

## Results

### Study selection and characterization

**Figure 1:**
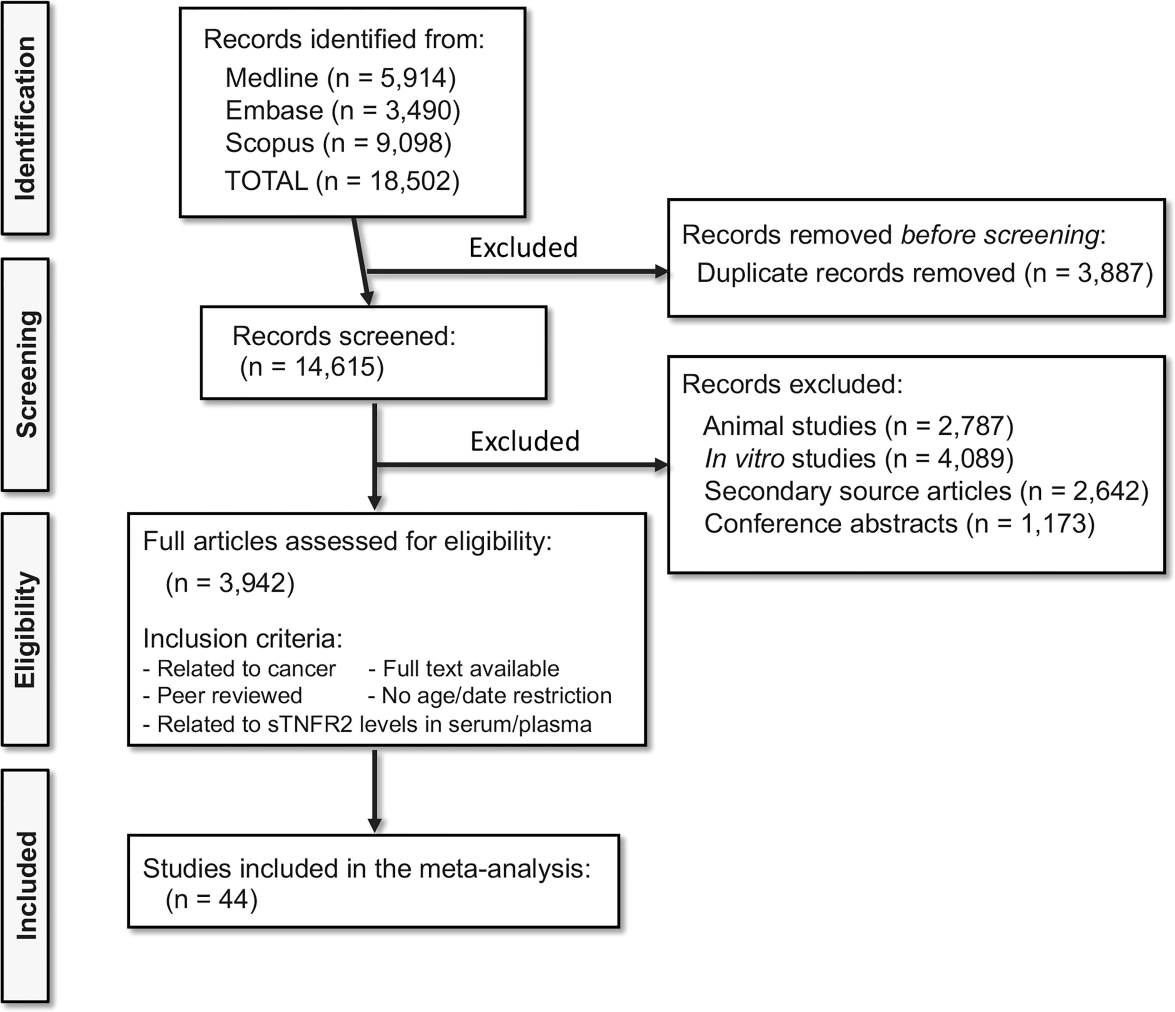
Selection of studies following PRISMA flowchart. Medline, Embase, and Scopus database searches resulted in the identification of 18,502 relevant articles. Following the exclusion and inclusion criteria, 44 articles were selected for the meta-analysis.

A total of 18,502 relevant articles were identified from Medline, Embase and Scopus searches. Of the 18,502 articles, 3,887 were duplicates and removed, and 14,615 records were selected for screening. After excluding 2,787 animal studies, 4,089 *in vitro* studies, 2,642 secondary source articles, and 1,173 conference articles, 3,942 full-length articles were assessed for their eligibility. After applying the inclusion and exclusion criteria, 44 studies (53–97) were included in this meta-analysis. PRISMA Flow chart (**Figure 1**) shows the number of studies searched and selected. The full list of the selected studies is listed in **Supplementary Table 1**.

### STROBE checklists

All the articles were assessed for their fitness following the STROBE checklist criteria. This includes meeting the criteria of participant selection, an introduction describing the background of the study, study design, the methods of sample handling, the results, and the outcome of the study. Each criterion scores one, and all the studies selected here were scored six and thus regarded as good quality studies for the purpose of this meta-analysis (**Supplementary Table 1**).

### sTNFR2 levels in the circulation of cancer patients

**Figure 2:**
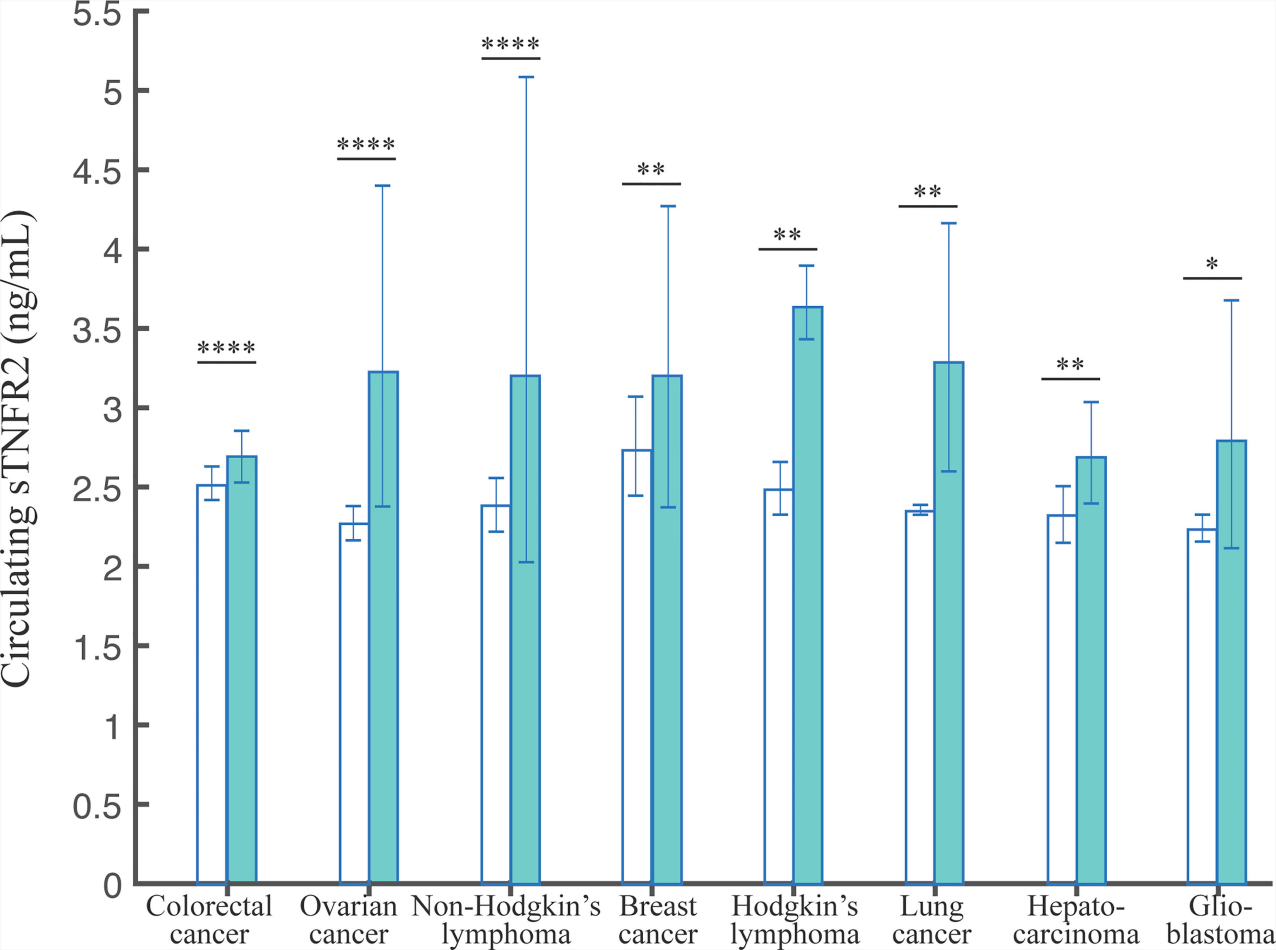
The pooled weighted means +/- 95% CI of sTNFR2 levels in serum/plasma from patients with the indicated cancers. The Tukey test was used to assess the significant difference of sTNFR2 levels, with *, **, and **** indicate P-values of <0.05, <0.01, <0.0001.

We investigated the evidence for differences in sTNFR2 levels in serum/plasma between healthy subjects and cancer patients, to explore its potential as a diagnostic biomarker. Here, we analyzed the cancer types that are observed by at least two studies (**Table 1**). In total, we extracted data from 28 studies, encompassing 7520 healthy and 5981 cancer participants. The pooled mean values of the healthy controls did not differ significantly across different cancers. On the contrary, we observed that the pooled mean of sTNFR2 in patients with colorectal cancer at 2.69 ng/mL (95% CI:2.45-2.95) was significantly higher (P-value of the difference <0.001) than that of the healthy controls at 2.51 ng/mL (95% CI:2.36-2.68) in the same studies. Similarly, the pooled mean of sTNFR2 in patients with ovarian cancer at 3.23 ng/mL (95% CI:2.28-4.59) was also significantly higher than that of the healthy controls (P-value of the difference <0.05) at 2.27 ng/mL (95% CI:2.15-2.40) in the same studies (**Table 1**). The significant differences in the levels of sTNFR2 in the serum/plasma between controls and cancer participants were also observed in several other cancers including non-Hodgkin’s lymphoma, breast cancer, Hodgkin’s lymphoma, lung cancer, hepatocarcinoma, and glioblastoma (**Figure 2**, **Table 1**). This result suggests the potential involvement of circulating sTNFR2 concentrations in cancer and could prove its utility as a diagnostic biomarker for cancer.

**Table 1:** The pooled weighted means of sTNFR2 levels in serum/plasma with 95% CI.

Cancer Type	No. of studies	No. of healthy controls	Pooled weighted mean of controls	95% CI	No. of cancer patients	Pooled weighted mean of cancer patients	95% CI	P-value
Colorectal cancer	9	5428	2.51	2.36- 2.68	3188	2.69	2.45- 2.95	< 0.0001
Ovarian cancer	5	594	2.27	2.15- 2.40	549	3.23	2.28- 4.59	< 0.0001
Non-Hodgkin’s lymphoma	5	851	2.38	2.19- 2.58	885	3.21	1.90- 5.43	< 0.0001
Breast cancer	3	325	2.74	2.48- 3.03	349	3.18	2.45- 4.13	0.0032
Hodgkin’s lymphoma	2	52	2.50	2.38-2.61	580	3.64	3.48- 3.81	0.0030
Lung cancer	2	57	2.36	2.33- 2.38	119	3.29	2.78- 3.90	0.0031
Hepato-carcinoma	2	213	2.32	2.19- 2.45	98	2.69	2.47- 2.93	0.0070
Glio-blastoma	2	191	2.24	2.18- 2.30	245	2.79	2.29- 3.41	0.0121

### sTNFR2 association with the risk of developing cancer

**Figure 3:**
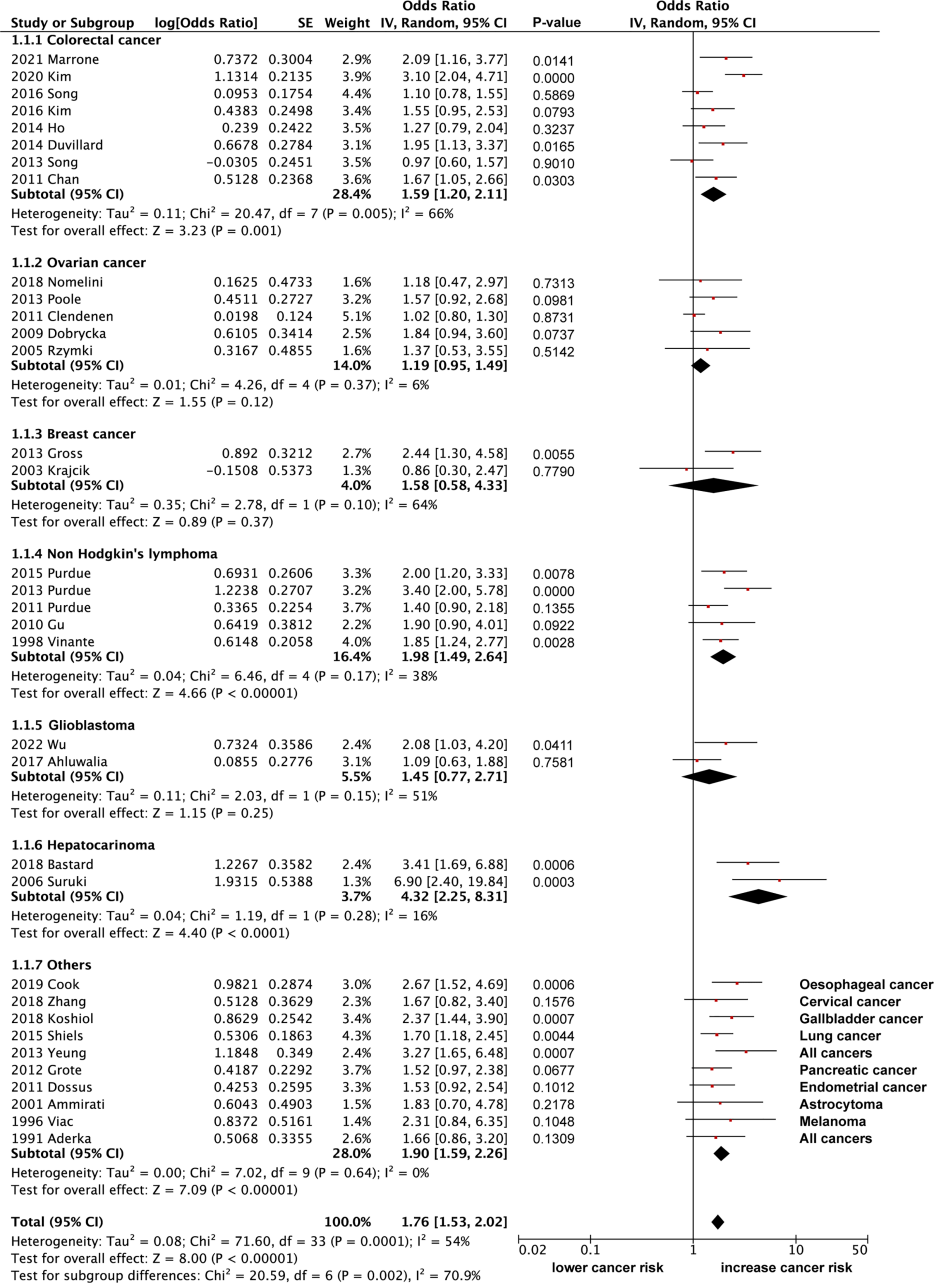
The forest plot showing overall OR of sTNFR2 and its correlation with cancer risk.

**Figure 4:**
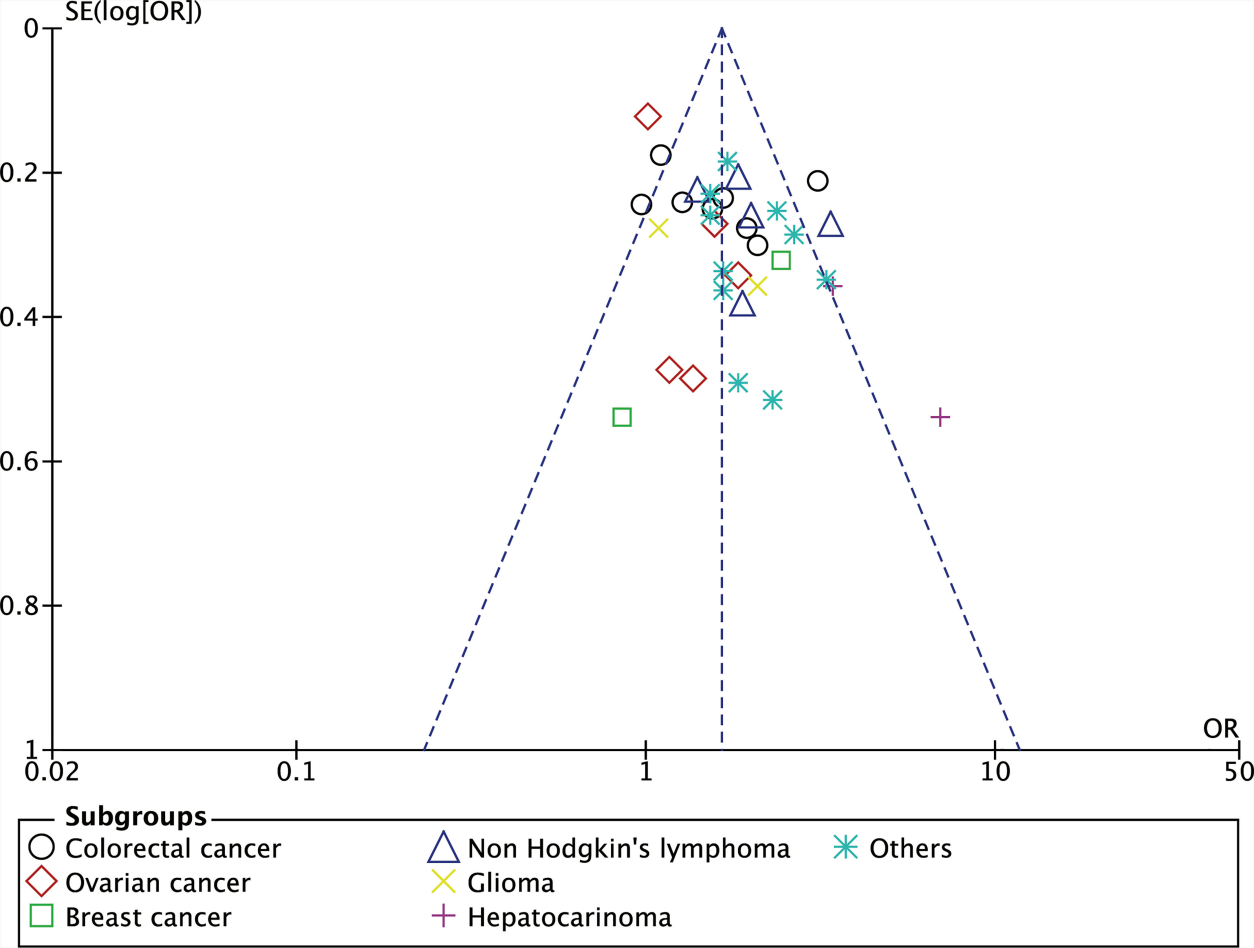
The funnel plot showing the OR of individuals studies against the standard error of the OR to detect bias and heterogeneity of between study.

Here, we extracted data from 34 eligible articles with sufficient data on the odd ratios (OR) for sTNFR2 and the risk of developing cancer (**Figure 3**). We divided the studies based on the cancer types that are being investigated and performed the random effect meta-analysis. Based on 8 studies, increased sTNFR2 levels showed significant association with colorectal cancer with pooled OR of 1.59 (95% CI:1.20-2.11), however significant heterogeneity of 66% between studies was also observed, although bias in heterogeneity could result from such a small number of studies (98). This heterogeneity result indicates that the strength of the association may differ between those studies. From 5 studies on non-Hodgkin’s lymphoma, increased sTNFR2 levels showed a significant association, with pooled OR of 1.98 (95% CI:1.49-2.64) and a non-significant heterogeneity of 38%. Additionally, 2 studies on hepatocarcinoma showed a strong significant association between sTNFR2 levels and this cancer, with pooled OR of 4.32 (95% CI:2.25-8.31) with a non-significant heterogeneity of 16%. On the other hand, studies with ovarian cancer, breast cancer, and glioblastoma did not show significant correlations between sTNFR levels and an increased risk in developing those cancers, with pooled OR of 1.19 (95% CI:0.95-1.49), 1.58 (95% CI:0.58-4.33), and 1.45 (95% CI:0.77-2.71) respectively. Overall pooled OR score from various cancers however showed a modest but significant correlation between sTNFR2 and an increased risk in cancer development, at 1.76 (95% CI:1.53-2.02), albeit with significant heterogeneity between studies of 54%. No publication bias was observed based on the funnel plot (**Figure 4**). These studies thus showed that sTNFR2 potentially increases the risk of some cancers to various extent, significantly in colorectal cancer, non-Hodgkin’s lymphoma, and hepatocarcinoma. This thus indicate sTNFR2 potential to be used as a circulating diagnostic biomarker for cancer.

We further tested our selected studies comprising various cancer types using a funnel plot, as this plot could indicate study heterogeneity and reporting bias. The funnel plot (**Figure 4**) shows a symmetrical feature which indicates the absence of reporting bias and that the random-effect model assumption used in this meta-analysis fits with the heterogeneity present in the selected studies (99).

## Discussion

Overall, most cancers still present with a high mortality rate, due to late diagnosis. As such, there is a pressing need for the identification of reliable biomarkers facilitating early detection. It has been frequently proposed that inflammation, orchestrated by various cytokines may promote cancer formation and further its development (6, 48). Cancer and immune cells may secrete immune proteins to control inflammation, such as sTNFR2, that may also mark cancer formation. Thus, in this systematic review, we investigated the correlation between increased circulating sTNFR2 levels with the risk of developing cancer. Finding the utility of sTNFR2 as a diagnostic marker could be useful to improve the effectiveness of current cancer diagnosis and provide a convenient detection approach for patients, especially with various technologies have been developed recently to facilitate circulating cytokine detection (100).

**Figure 5:**
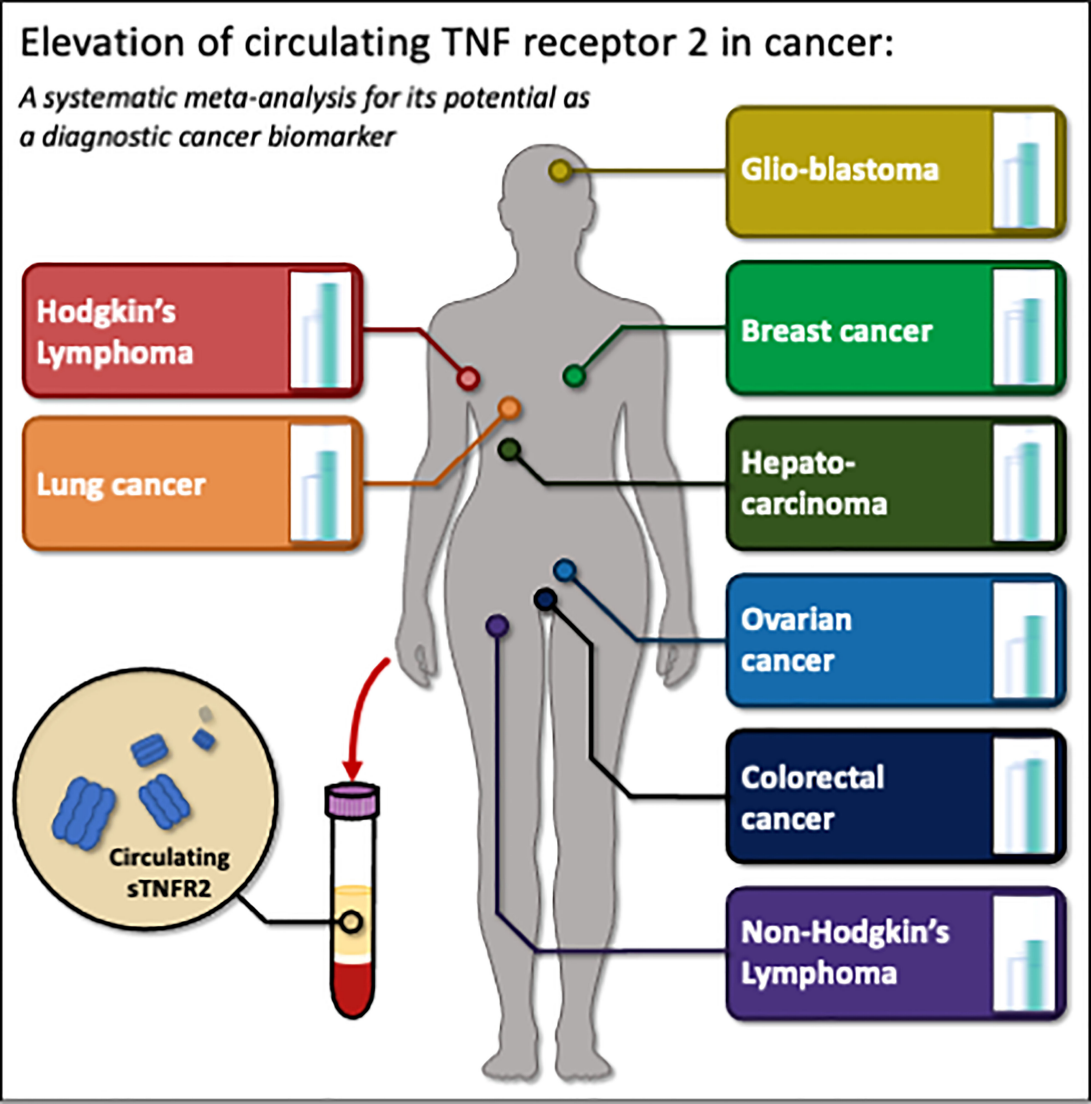
The summary schema of the elevated sTNFR2 levels in various cancers, and its potential as a biomarker for cancer detection. Figure was created using Microsoft power point.

Based on the calculated pooled mean values, circulating sTNFR2 levels were found to be consistently reported as significantly higher in various cancers in comparison to the healthy controls (**Figure 5**), suggesting sTNFR2 may be involved in cancer development. Using a random-effect OR meta-analysis, we observed significant correlations between sTNFR2 and several cancers, including colorectal cancers, non-Hodgkin’s lymphoma, and hepatocarcinoma. This indicates the potential of sTNFR2 as a diagnostic biomarker. Indeed, sTNFR2 levels are correlated with lung cancer development even 6 years before diagnosis (84). However, in other cancers, including ovarian, breast, and glioblastoma, the correlation was not significant, suggesting that sTNFR2 levels may not be sufficient as an independent diagnostic biomarker, especially for these cancers. It has been previously suggested that the circulating inflammatory biomarkers such as sTNFR2 which are highly correlated with cancer risks, could be combined with the circulating cancer-specific biomarkers that otherwise would have low sensitivity and specificity to indicate the presence of cancer (100). Furthermore, in agreement with our findings, a previous meta-analysis of prospective studies also found no significant association (of diagnostic value) between circulating levels of sTNFR2 and the risk of ovarian cancer (101). However, some studies show that high expression of TNFR2 at cancer sites is correlated with cancer size, metastasis and progression in epithelial ovarian cancer (90, 102), non-small lung carcinoma (103), anal carcinoma (104), and esophageal carcinoma (105, 106).

The increased levels of circulating sTNFR2 do not only correlate with cancer risk but also cancer outcomes such as overall survival and progression-free survival, as has been shown by several studies of various cancers (56, 65–67, 78, 88, 107–113). In patients with ovarian cancer for example high levels of TNFR2*^+^* Tregs have been associated with poor OS, while ovarian tissue with strong expression of TNFR2 was associated with longer PFS (14, 114). Additionally, a high pre-diagnosis plasma sTNFR2 level corelate with overall mortality in colorectal cancer patients (110). Moreover, several studies show that the effectiveness of anti-cancer drugs at reducing tumor size and improving survival is correlated with reduced levels of circulating sTNFR2 (115–119). This observation may thus extend the use of sTNFR2 not only as a minimally-invasive diagnostic biomarker to predict cancer outcome, but also to monitor therapy effectiveness during treatment (120).

In summary, our meta-analysis study confirms the correlation between increased circulating sTNFR2 levels and increased risk of cancers, albeit the extent of this association varies between different cancers. This indicates circulating sTNFR2 may have utility, perhaps combined with other blood accessible biomarkers, to aid in the diagnosis of cancer. We believe that the easy access to this biomarker through liquid biopsies makes it an ideal candidate to be used alone, or in combination with other markers, as a minimally-invasive cancer screening method, potentially accelerating the implementation of point-of-care devices (100) for cancer diagnosis in clinical settings outside of central laboratories.

## Data availability statement

The raw data supporting the conclusions of this article will be made available by the authors, without undue reservation.

## Author contributions

AK, MP conceptualized the study. AK designed and directed the study. AK, MR and EC performed literature research and analyzed the data. AK wrote the first draft of the manuscript. EC, MR, CS, CH, AM, MP wrote sections of the manuscript. All authors contributed to the article and approved the submitted version.

## Funding

This research was funded by NHMRC No. 2011747.

## Conflict of interest

The authors declare that the research was conducted in the absence of any commercial or financial relationships that could be construed as a potential conflict of interest.

## Publisher’s note

All claims expressed in this article are solely those of the authors and do not necessarily represent those of their affiliated organizations, or those of the publisher, the editors and the reviewers. Any product that may be evaluated in this article, or claim that may be made by its manufacturer, is not guaranteed or endorsed by the publisher.
